# Risk of recurrent subarachnoid haemorrhage, death, or dependence and standardised mortality ratios after clipping or coiling of an intracranial aneurysm in the International Subarachnoid Aneurysm Trial (ISAT): long-term follow-up

**DOI:** 10.1016/S1474-4422(09)70080-8

**Published:** 2009-05

**Authors:** Andrew J Molyneux, Richard SC Kerr, Jacqueline Birks, Najib Ramzi, Julia Yarnold, Mary Sneade, Joan Rischmiller, for the ISAT collaborators

**Affiliations:** a Neurovascular Research Unit, Nuffield Department of Surgery, University of Oxford and Oxford Radcliffe Hospitals NHS Trust, John Radcliffe Hospital, Oxford, UK; b Centre for Statistics in Medicine, Oxford, UK

## Abstract

**Background:**

Our aim was to assess the long-term risks of death, disability, and rebleeding in patients randomly assigned to clipping or endovascular coiling after rupture of an intracranial aneurysm in the follow-up of the International Subarachnoid Aneurysm Trial (ISAT).

**Methods:**

2143 patients with ruptured intracranial aneurysms were enrolled between 1994 and 2002 at 43 neurosurgical centres and randomly assigned to clipping or coiling. Clinical outcomes at 1 year have been previously reported. All UK and some non-UK centres continued long-term follow-up of 2004 patients enrolled in the original cohort. Annual follow-up has been done for a minimum of 6 years and a maximum of 14 years (mean follow-up 9 years). All deaths and rebleeding events were recorded. Analysis of rebleeding was by allocation and by treatment received. ISAT is registered, number ISRCTN49866681.

**Findings:**

24 rebleeds had occurred more than 1 year after treatment. Of these, 13 were from the treated aneurysm (ten in the coiling group and three in the clipping group; log rank p=0·06 by intention-to-treat analysis). There were 8447 person-years of follow-up in the coiling group and 8177 person-years of follow-up in the clipping group. Four rebleeds occurred from a pre-existing aneurysm and six from new aneurysms. At 5 years, 11% (112 of 1046) of the patients in the endovascular group and 14% (144 of 1041) of the patients in the neurosurgical group had died (log-rank p=0·03). The risk of death at 5 years was significantly lower in the coiling group than in the clipping group (relative risk 0·77, 95% CI 0·61–0·98; p=0·03), but the proportion of survivors at 5 years who were independent did not differ between the two groups: endovascular 83% (626 of 755) and neurosurgical 82% (584 of 713). The standardised mortality rate, conditional on survival at 1 year, was increased for patients treated for ruptured aneurysms compared with the general population (1·57, 95% CI 1·32–1·82; p<0·0001).

**Interpretation:**

There was an increased risk of recurrent bleeding from a coiled aneurysm compared with a clipped aneurysm, but the risks were small. The risk of death at 5 years was significantly lower in the coiled group than it was in the clipped group. The standardised mortality rate for patients treated for ruptured aneurysms was increased compared with the general population.

**Funding:**

UK Medical Research Council.

## Introduction

Endovascular coiling for the treatment of intracranial aneurysms was first introduced into clinical use in 1990. A prospective randomised trial, the International Subarachnoid Aneurysm Trial (ISAT), which was supported by the Medical Research Council (MRC), was commenced as a pilot study in 1994 and ceased recruitment in 2002. 2143 patients with a recently ruptured intracranial aneurysm were enrolled in 43 centres and were randomly assigned to neurosurgical clipping or endovascular coiling. The trial protocol, methods, and results at 1 year have been published.^1^, ^2^, ^3^ The results demonstrated that coiling was associated with a reduction in the risk of death and dependency at 1 year, which was the primary outcome: 250 (24%) of 1063 patients randomly assigned to coiling were dead or dependent compared with 326 (31%) of 1055 patients randomly assigned to clipping, a relative risk reduction in death or dependence at 1 year of 23·9% (95% CI 12·4–33·9) and an absolute risk reduction of 7·4% (3·6–11·2; p=0·0001).

Since the introduction of coiling of cerebral aneurysms, there have been concerns about the durability of coil treatment and its ability to prevent subsequent rebleeding of the treated aneurysm. This is particularly important because many individuals with ruptured intracranial aneurysms are young; the mean age at entry into the trial was 52 years. Thus, patients who survive in good clinical condition following treatment have a potentially long life expectancy. The angiographic occlusion rates of coiling are worse than the rates after surgical clipping,^4^, ^5^, ^6^ and a higher proportion of patients required retreatment of a previously coiled aneurysm.^4^ The limited long-term follow-up data after coiling was acknowledged in both our earlier papers and has been emphasised throughout the published works.^7^, ^8^

In addition, evidence is emerging that patients who have an initial subarachnoid haemorrhage (SAH) from an aneurysm are at greater risk of the formation of a new aneurysm and a further SAH compared with the general population;^9^, ^10^ furthermore, they might have a higher overall standardised risk of death compared with the age-matched and sex-matched population.^11^

One of the original objectives of ISAT, which was set out in the protocol, was to continue long-term follow-up of the randomised patients in a systematic manner in an attempt to answer these questions. We continue to follow up all UK patients, and some Scandinavian and Canadian centres follow up patients in their respective countries. This has provided a unique opportunity to observe, prospectively, in a well-defined population who have had clipping or coiling, the risk of further subarachnoid haemorrhage and other related complications, including long-term risk of death, after treatment of a ruptured cerebral aneurysm.

## Methods

### Patients

The trial methodology was described in detail in the protocol and has been published elsewhere with the inclusion criteria, ethics approval, and consent criteria.^1^, ^2^ Table 1 shows the baseline characteristics.

**Table 1:** Baseline characteristics

		**Endovascular (n=1073)**	**Neurosurgery (n=1070)**
Men		400 (37%)	398 (37%)
Age (years)		52 (44–60 [18–87])	52 (43–60 [18–84])
WFNS grade			
	1	674 (63%)	661 (62%)
	2	269 (25%)	280 (26%)
	3	66 (6%)	68 (6%)
	4	38 (4%)	36 (3%)
	5	11 (1%)	9 (1%)
	6 (not assessable)	15 (1%)	16 (1%)
Maximum size of the lumen of the target aneurysm			
	≤5 mm	553 (52%)	572 (53%)
	6–10 mm	437 (41%)	426 (40%)
	≥11 mm	83 (8%)	72 (7%)
Number of aneurysms			
	1	836 (78%)	850 (79%)
	2	173 (16%)	170 (16%)
	3	44 (4%)	35 (3%)
	≥4	20 (2%)	15 (1%)
Time between SAH and randomisation (days)		2 (1–4 [0–26])	2 (1–5 [0–28])

Data are number (%) or median (IQR [range]). WFNS= World Federation of Neurosurgical Societies. SAH=subarachnoid haemorrhage.

### Procedures

The centres that are undertaking long-term follow-up enrolled 2004 patients of the original cohort of 2143. The patients receive annual postal questionnaires concerning their dependency, quality of life (EUROQOL [EQ5D]), and whether they have had any further hospital admissions or further treatment related to their SAH or aneurysm. All UK patients are flagged with the Office for National Statistics (ONS), and the Oxford Neurovascular and Neuroradiology Research Unit (ONNRU) automatically receives notification of the death of all patients in the study who have died in the UK. Suspected instances of rebleeding are either brought to the attention of investigators by the centre, self-reported by the patient or their family, or identified when notified by ONS to ONNRU; the same is true of any further hospital admissions. The data in respect of any rebleeds are collected, including the date, any operative reports, post-mortem reports, and imaging data. These were reviewed by an experienced neurosurgeon (RSCK, NR) and neuroradiologist (AJM). All centres followed-up patients annually for 5 years. All UK and eight non-UK centres continue to follow-up patients annually.

### Statistical analysis

Survival analyses were done for time to rebleeding and time to death, measured from the time of the initial SAH. For the analysis of time to rebleeding, observations were censored when a patient was lost to follow-up or had died. For the analysis of time to death, observations were censored when a patient was lost to follow-up. Data were retrieved for up to 5 years for all UK and non-UK deaths and for more than 5 years in all UK centres and those non-UK centres that continued follow-up. Data for the survival and rebleed analyses were extracted on Aug 31, 2008. Kaplan–Meier curves were calculated for each treatment group, and the groups were compared with the log-rank test. We calculated Kaplan–Meier curves for the rebleeding events on the basis of allocated group and by actual treatment received. The analyses were performed with Stata, version 10 (StatCorp LP, TX, USA).

To calculate the standardised mortality ratios (SMRs) all patients' deaths were included from a date 1 year from the date of their SAH until the end of 2007. Standard death rates for England and Wales were used, which covered 5-year age bands from 15 until 25 years, 10-year age bands until 85 years of age, and one age band for over 85 years, for all years from 1998 until 2007. The number of patients alive on the first day of each year, at least 1 year from the date of their SAH was ascertained, and the number of deaths during the year was counted. The age of each patient was assessed as though at the midpoint of each year. The expected number of deaths was calculated for each year and summed over all years. ISAT is registered, number ISRCTN49866681.

### Role of the funding source

The sponsor had no role in the study design, data collection, data analysis, data interpretation, or the writing of the report. The principal investigators (RSCK and AJM) had full access to all the data in the study and had final responsibility for the decision to submit for publication.

## Results

**Figure 1:**
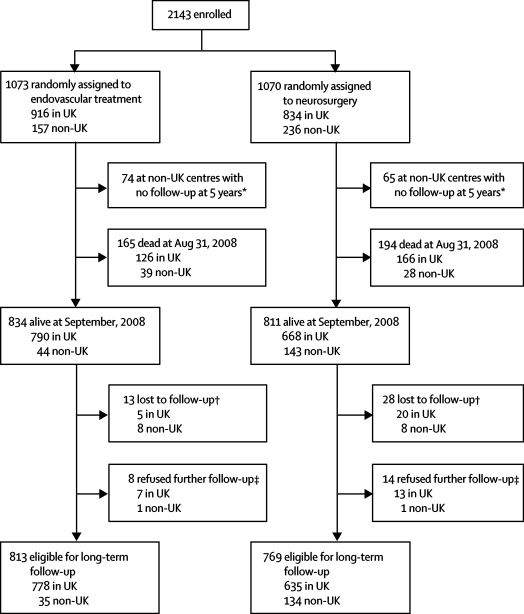
Study profile *Some non-UK centres no longer follow-up patients. †Lost patients had moved abroad (from country of randomisation) or had had no contact with study centre or primary-care physician since the first or second year of study. ‡Patient has withdrawn or they no longer wish to participate.

Figure 1 shows the study profile. Complete loss to follow-up was 2·7% at a mean of 9 years. As of August, 2008, there were 1307 survivors in the UK and 275 survivors in non-UK centres who continued follow-up; some non-UK centres ceased follow-up after 5 years (late non-UK follow-up is unfunded). We report these data to a minimum of 6 years and a maximum of 14 years after enrolment in the trial.

**Figure 2:**
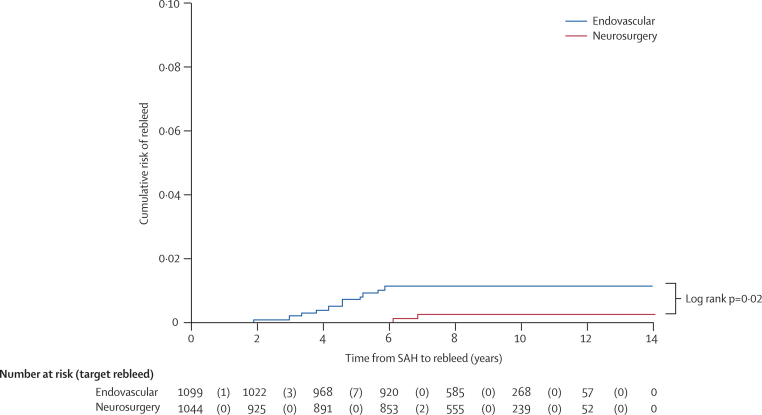
Kaplan–Meier graph of cumulative risk of rebleed from the treated aneurysm after more than 1 year by treatment allocation Patients followed up for a minimum of 6 years and a maximum of 14 years. SAH=subarachnoid haemorrhage.

Table 2 shows the confirmed rebleeding (recurrent SAH) events. 24 rebleeds have been confirmed in 24 patients. In 13 patients, the rebleeding was from the treated aneurysm or in the same location as the previously treated aneurysm, but in ten patients the bleeding occurred from either a new aneurysm or a pre-existing aneurysm that had not previously ruptured. In one patient the source of the haemorrhage was unknown. In four patients the bleeding occurred from another aneurysm that was seen at the original presentation, and in six patients the bleeding was confirmed as arising from a new (de novo) aneurysm, which had not been present on the angiogram at the time of the original presentation. Figure 2 shows the Kaplan–Meier curve of time to rebleed from the target aneurysm by the treatment received.

**Table 2:** Rates of recurrent SAH after more than 1 year by treatment allocation

	Rebleeding from target aneurysm*	**Rebleeding from aneurysm that was known at baseline**†	**De novo aneurysm**‡	**Unknown aneurysm**	**Total**
Endovascular (8447 person-years)	10 (3)	3 (2)	3 (1)	1 (1)	17 (7)
Neurosurgery (8177 person-years)	3 (3)§	1 (1)	3 (2)	0	7 (6)
Total	13 (6)	4 (3)	6 (3)	1 (1)	24 (13)

Numbers in parenthesis are deaths within 30 days of bleeding.

* The target aneurysm is identified at the time of enrolment in the trial.

† Other known aneurysms that were seen on the first angiogram but are not thought to have ruptured.

‡ De novo aneurysms that were not seen on the first angiogram.

§ One patient crossed over to coiling. SAH=subarachnoid haemorrhage.

In the endovascular cohort, there were ten episodes of rebleeding from the treated aneurysm after 1 year in 8447 person-years of follow-up. In these ten patients the rebleeding arose from the aneurysm that had been treated after SAH. Of these ten patients, three died within 30 days of the second bleed, two were disabled after rebleed and retreatment (mRS scores of 4 or 5 at follow-up), and four patients were retreated and were independent at follow-up, although one of these died 2 years later of cancer. One patient rebled 2 days after a second aneurysm coiling for a neck remnant but did not require any further treatment and was independent at follow-up.

In three patients, rebleeding was shown to occur from another known aneurysm, and in another three patients, rebleeding occurred from a new aneurysm that was not present on the original angiogram. In one patient, it was impossible to establish the source of the recurrent SAH.

In the neurosurgical cohort, three patients had a further subarachnoid haemorrhage from the treated aneurysm after 1 year in 8177 person-years of follow-up; all three patients died within 30 days of the rebleed. One of these patients, who had an anterior communicating artery aneurysm, had declined surgery after randomisation and underwent coiling of the aneurysm. The aneurysm was incompletely occluded at follow-up angiography, but the patient declined further treatment, and rebled in year 4. The other two patients underwent surgical clipping of the ruptured aneurysm (a middle cerebral and an anterior communicating artery aneurysm, respectively). Occlusion of the aneurysms was judged complete at surgery in both, but no early or late postoperative angiography was done. The patients rebled and died at 6·5 years and 7 years, respectively. One patient bled from another known aneurysm, and three patients bled from a new aneurysm that was not present at the time of the original angiogram. The time to recurrent SAH from the treatment of the aneurysm in the endovascular group was between 2 and 5 years. In the surgical group, the time to recurrent SAH was between 4 and 7 years (figure 2). There was a non-significant increased risk of rebleeding from the treated aneurysm in the endovascular cohort (log rank p=0·06) by intention-to-treat analysis and a significant difference when analysis was by actual treatment received (log rank p=0·02). There was no difference between the groups in mortality rates as a result of rebleeding. The time to recurrent haemorrhage from pre-existing aneurysms was between 2 and 4 years, and that from new aneurysms was between 4 and 9 years.

**Figure 3:**
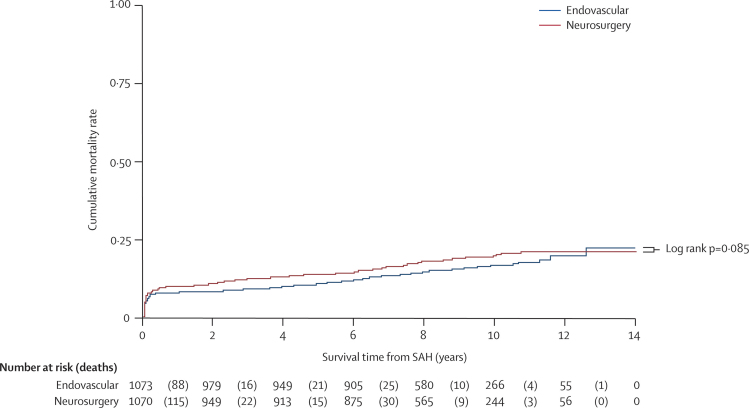
Kaplan–Meier graph of cumulative mortality rate Patients followed up for a minimum of 6 years and a maximum of 14 years. SAH=subarachnoid haemorrhage.

The number of deaths reported to the end of August, 2008, was 359. The predominant causes of death after 1 year in both groups were cancer or cardiovascular disease and these were listed in our previous report.^2^ Case-fatality rates at 1 year were 8·0% (95% CI 6·4–9·8) and 9·9% (8·2–11·0) among patients allocated to endovascular treatment and neurosurgery, respectively. At 5 years, 112 of 1046 (11%) and 144 of 1041 (14%) patients were dead among those allocated to the endovascular and neurosurgical treatment groups, respectively (odds ratio [OR] 0·75, 95% CI 0·58–0·97; p=0·03). Figure 3 shows the Kaplan–Meier curves for cumulative mortality and numbers at risk at seven time points. Table 3 shows the SMRs of the UK cohort, conditional on 1 year survival after the SAH and based on deaths to the end of 2007. There have been a further 91 deaths since the preparation of the data in 2004: 35 due to cancer, 21 due to cardiovascular disease, eight due to respiratory disease, five due to ischaemic stroke, and two probable suicides. Six deaths were due to recurrent SAH: five from another aneurysm and one from the treated aneurysm (a clipped patient). 12 deaths were due to various causes that were unrelated to the SAH; the causes of two deaths are unknown.

**Table 3:** Standardised mortality ratios calculated for UK cohort, conditional on survival at 1 year

	**Observed deaths**	**Expected deaths**	**SMR**	**p**
All	144	92	1·57 (1·32–1·82)	<0·0001
Female	90	55	1·65 (1·32–1·98)	<0·0001
Male	54	37	1·46 (1·09–1·83)	0·02
Endovascular	63	46	1·37 (1·04–1·70)	0·03
Neurosurgery	81	46	1·77 (1·00–2·14)	<0·0001

Data are number or SMR (95% CI). Ratios were calculated for the UK cohort conditional on survival at 1 year. SMR=standardised mortality ratio.

The primary outcome of the trial was death or dependency. At 5 years, mRS scores were available for 867 of 1073 patients in the endovascular group and 857 of 1070 patients in the neurosurgical group. 41 patients (2%) were completely lost to follow-up. The mRS score for dependency at the 5-year follow-up was missing for 178 patients in the endovascular group and 184 patients in the neurosurgical group, but their vital status was known. The number of missing mRS scores did not differ between the groups; in most patients values were available for earlier or later years. The proportion of patients with an mRS of 2 or less (ie, independent) conditional on survival at 5 years, was 626 of 755 (83%) in the endovascular group and 584 of 713 (82%) in the neurosurgical group; mRS scores were also available at other time points before and after 5 years, but a longitudinal analysis of these data is beyond the scope of this paper. Table 4 shows the mRS scores available at 5 years in both groups.

**Table 4:** Clinical outcomes at 5 years

		Endovascular (n=1046*; n=867)†	Neurosurgery (n=1041*; n=857)†
mRS score			
	0 (no symptoms)	264	198
	1 (minor symptoms)	217	211
	2 (some restriction in lifestyle)	145	175
	3 (substantial restriction in lifestyle)	83	93
	4 (partly dependent)	24	18
	5 (fully dependent)	22	18
	6 (dead)	112	144
	0–2 inclusive	626	584
	3–6 inclusive	241	273
Probability of independence conditional on survival at 5 years		626 of 755 (83%)	584 of 713 (82%)
Probability of death		112 of 1046 (11%)	144 of 1041 (14%)
Relative risk of non-independence conditional on survival at 5 years		0·99, 0·94–1·03, p=0·61	
Relative risk of death at 5 years		0·77, 0·61–0·98, p=0·03	
Probability of survival and independence at 5 years		74%	71%

Data are number; number (%); relative risk, 95% CI, p value; or percentage. mRS=modified Rankin scale.

* Ascertainment for death was almost complete but dependency status was missing (n=27 for endovascular; n=29 for neurosurgery).

† Incomplete ascertainment of mRS at 5 years (n=206 missing for endovascular; n=213 missing for neurosurgery). Reasons for missing mRS score: centre did not follow up patients (n=56); mRS not available, data temporarily or permanently missing, or no dependency outcome value given at year 5 (n=418).

## Discussion

There is continuing concern, particularly in the neurosurgical community, that the initial observed clinical benefit of coiling compared with clipping could be lost over time if subsequent rebleeding rates are high. The data we now report are reassuring. Although, on the basis on these data, the annual risk of the treated aneurysm rebleeding is higher in the patients treated by coiling than in the patients treated by clipping, the risk remains low and is at a similar level to the risk of further SAH from another source, either a pre-existing aneurysm or a newly formed aneurysm. Until recently, neurosurgeons had assumed that the risk of a recurrent SAH after clipping of an aneurysm was close to zero. Recent data from Wermer and coauthors^9^ on the late risk of haemorrhage in a large cohort of patients who previously had their aneurysms clipped showed a significantly increased risk of recurrent SAH compared with the general population (22 times [95% CI 12–38] compared with age-matched and sex-matched population).^9^ Wermer and coauthors also found the prevalence of aneurysms to be 17% on follow-up CT angiography.^12^ The data we report here appear to support this finding and suggest that the previous assumptions that the risk of recurrent SAH was similar to that in the general population were incorrect. The baseline characteristics of the ISAT cohort were similar in terms of sex, age at the time of the haemorrhage, and proportion of patients with several aneurysms.

Of the patients who have a SAH from a ruptured aneurysm, about 20% have more than one aneurysm at the time of presentation and have a continuing risk of developing new aneurysms. Smoking and female gender are known risk factors for new aneurysm formation and SAH; unfortunately, smoking history was not collected during the trial. The timing of late recurrent haemorrhage is noteworthy; all rebleeding events from the coiled aneurysms have so far occurred within 5 years of the initial event, whereas the late recurrent haemorrhages that have occurred after 5 years have been from another aneurysm or, in one patient, a clipped aneurysm. This raises the possibility that the risk of a recurrent haemorrhage from a treated aneurysm might not be constant over time; however, a substantially longer observation period would be needed to confirm if this is the case.

Our findings confirm those of Wermer and coauthors that patients who have had treatment for a ruptured aneurysm have a small but definitely increased risk of recurrent SAH compared with the general population. The late all-cause mortality risk is also increased compared with the general population, and the SMR in the surviving UK population is significantly increased (p<0·0001). This raises the important issue of how best to follow up patients with coiled or clipped aneurysms. In recent years, there has been a rapid shift from the use of invasive angiography in the follow-up of patients with coiled aneurysms to the use of non-invasive MRI techniques, such as magnetic resonance angiography (MRA), contrast-enhanced MRA, and CT angiography (CTA) in patients with clipped aneurysms. These imaging methods are much more acceptable to patients and involve no angiographic stroke risk; however, any follow-up screening for the recurrence of new aneurysms has substantial health-care costs and the potential for substantial psychological morbidity and anxiety.^13^ What is of considerable importance is strong advice on smoking cessation and the proper management of hypertension and other cardiovascular risk factors.

The question asked in the neurosurgical community about coiling has always been: will the early proven benefit of coiling be lost in the long term? This question was highlighted and modelled in a recent paper and associated commentary.^14^, ^15^ This paper has given rise to controversy, particularly with respect to the management of young patients with ruptured aneurysms (younger than 40 years, who were only a small subgroup in the trial).^14^

The level of risk we now report and the absence of an increase in risk of death between the patients treated with coiling and those treated with clipping over what is now a mean of 9 years follow-up would appear not to change the message of our original paper; namely, that for patients with suitable aneurysms, coiling is more likely than clipping to result in improved clinical outcomes at 1 year, and these data suggest that although the early clinical benefits are reduced over time, they are not lost over the subsequent 4 years. We have previously reported^4^ that patients undergoing coiling were more likely to require delayed aneurysm retreatment. Late retreatment (more than 3 months after first treatment) was done in 9% of patients who had coiling, a hazard ratio of 6·9 (95% CI 3·4–14·1) compared with clipping. However, there was no change in mRS score at follow-up after further treatment; thus, the further treatment after initial coiling had no effect on the 5-year outcomes.^4^ At 5 years, there is a significantly reduced risk of death in the endovascular group compared with the neurosurgical group. For those patients who are alive at 5 years, there is no significant difference in the probability of being independent, which appears to indicate the continuing improvement in the functional outcome of less severely disabled patients in the surgical group between year 2 and year 5. One explanation for this might be an increased case-fatality rate among the more severely dependent patients who had neurosurgery.

The inferences from all these reports are the same: the extrapolation of the results of any clinical trial to individual patients must be done after weighing all the factors of an individual's circumstances, the health-care environment, the equipment available, the availability of relevant neurosurgical and endovascular skills, the anatomy and location of the aneurysm and the relative difficulties for the endovascular or surgical approach in the specific case, and the age and clinical state of the patient. These are all factors that could affect the advice that might be provided to patients and their relatives. This emphasises the need for patients with such a complicated care pathway to be managed at centres where both treatment options are available.

When ISAT started recruiting patients in 1994, there were understandable questions about the change in technology that might occur in the interim and thus the relevance to clinical practice when such an inevitably long trial is completed. Patients were enrolled in ISAT during an early phase in the use of detachable coils; the first patient was enrolled only 2·5 years after the device was approved for clinical use in Europe and before the US Food and Drug Administration (FDA) gave approval. At that time, the range and performance of detachable coils and their associated catheters were limited. Considerable advances have taken place in the technical performance of coils and catheters, and in operator experience, technical expertise, and digital radiographic imaging systems, particularly three-dimensional angiography, which have all substantially improved since the trial was started. These developments might have reduced the risks of coiling; certainly, it seems unlikely that the current procedural or rebleeding risks are greater than those observed during the trial and the follow-up. The results of three other randomised studies that have recently completed recruitment—the Barrow Randomised Aneurysm Trial (BRAT) of coiling compared with clipping in ruptured aneurysm at a large US neurosurgical centre; the HydroCoil Endovascular Aneurysm Occlusion and Packing Study (HELPS) to compare hydrogel-coated coils with bare platinum coils;^16^ and the Cerecyte Coil Trial to compare polymer-loaded coils with standard platinum coils—will provide invaluable data on the current early clinical outcomes for patients undergoing coiling of a ruptured aneurysm. Further observation of the ISAT cohort will continue until at least 2013.

The ISAT follow-up for a mean of 9 years (range 6–14 years) demonstrates that the risk of rebleeding from a treated aneurysm is low. There were more rebleeds from the treated aneurysm in the coiling group than in the clipping group, but there was no difference between the groups in the number of deaths due to rebleeding. The risk of death at 5 years was significantly lower in the coiled group than it was in the clipping group. The probability of independent survival for those patients alive at 5 years is the same in the two groups. The SMR, conditional on survival at 1 year, is increased in patients treated for ruptured aneurysms compared with the general population.
